# Serum levels of miR-21-5p and miR-339-5p associate with occupational trichloroethylene hypersensitivity syndrome

**DOI:** 10.1186/s12995-021-00308-0

**Published:** 2021-05-17

**Authors:** Wei Liu, Jian Zheng, Xiaohu Ren, Yuxuan Xie, Dafeng Lin, Peimao Li, Yuan Lv, Maggie Pui Man Hoi, Yanfang Zhang, Jianjun Liu

**Affiliations:** 1 Shenzhen Key Laboratory of Modern Toxicology, Shenzhen Medical Key Discipline of Health Toxicology (2020-2024), Shenzhen Center for Disease Control and Prevention, Shenzhen, 518055 China; 2 Shenzhen Prevention and Treatment Center for Occupational Diseases, Shenzhen, 518020 China; 3 Key Laboratory of Molecular Epidemiology of Hunan Province, School of Medicine, Hunan Normal University, Changsha, 410081 China; 4 State Key Laboratory of Quality Research in Chinese Medicine, Institute of Chinese Medical Sciences, University of Macau, Macau, China

**Keywords:** Trichloroethylene, Trichloroethylene hypersensitivity syndrome, miRNAs, Biomarkers, Diagnostic model

## Abstract

**Background:**

Trichloroethylene (TCE) hypersensitivity syndrome (THS) is a dose-independent and potentially life-threatening disease. In this study, we sought to identify THS-related miRNAs and evaluate its potential clinical value.

**Methods:**

Serum samples of five patients and five matched TCE contacts were used for screening differential miRNAs. Another 34 patients and 34 matched TCE contacts were used for verifying significantly differential miRNAs with SYBR™ Green PCR and MGB PCR. The diagnostic model based on these miRNAs was established via the support vector machine (SVM) algorithm. Correlation between differential miRNAs and liver function was analyzed via the Spearman correlation test.

**Results:**

A total of 69 miRNAs was found to be differentially expressed. MiR-21-5p and miR-339-5p were verified to have significant higher expressions in patients. The sensitivity, specificity and accuracy of disease model were 100, 75 and 86%, respectively. The two miRNAs showed significant correlations with liver function.

**Conclusion:**

These findings suggested that miRNAs profiles in serum of THS patients had changed significantly, and miR-21-5p and miR-339-5p were associated with THS.

**Supplementary Information:**

The online version contains supplementary material available at 10.1186/s12995-021-00308-0.

## Introduction

Trichloroethylene (TCE) is a commonly used organic solvent used in refrigerant raw materials, metal cleaning raw materials, and for other purposes [1]. Its use in China has increased rapidly since the early 1990s in proportion to industrial growth of metal, electronics, and telecommunications. TCE also is an environmental contaminant that persists in the air, soil, and water [2, 3]. The U.S. Agency for Toxic Substances and Disease Registry (ATSDR) found that TCE from soil, water and air can enter indoor air and present a health hazard [4]. Strong, clear associations were observed between indoor air TCE concentrations and people’s blood TCE concentrations, as well as between soil gas TCE concentrations and blood TCE concentrations [5]. A recent study indicated that female MRL^+/+^ mice exposed to TCE at approximately environment levels (0.05 mg/L) produced lobular inflammation in the liver [6]. TCE as a water pollutant can alter gene-specific DNA methylation in CD4^+^ T cells of autoimmune disease [7].

The use of TCE in the factory poses a threat to workers’ health [8]. Both the U.S. Environmental Protection Agency (EPA) and the International Agency for Research on Cancer (IARC) classify TCE as a human carcinogen [9] (http://www.epa.gov/IRIS). Overexposure to TCE can trigger headache, dizziness, sleepiness, coma and even death. Occupational TCE exposure has been associated with dysfunction of cranial nerves, notably the trigeminal nerve (U.S. EPA, Toxicological Review of Trichloroethylene Appendix D. (CAS No. 79–01-6) EPA/635/R-09/011F, 2011). Occupational TCE poisoning incidents are commonly reported, of which trichloroethylene hypersensitivity syndrome (THS) is in the majority in China. THS is a T cell-mediated autoimmune disorder with potential for widespread effects, including liver, kidney and skin injury, and subsequently organ failure [10, 11]. For TCE-induced dermatitis, the average time of onset is about 28 days [12]. Over 500 cases have been reported since the first patient was found in Guangdong, China. The case fatality rate is about 9–13% [10]. According to the Guangdong provincial bureau of population statistics, more than a million people in the Province have passive contact with TCE. However, effective approaches for disease diagnosis and surveillance remain to be developed. One of the best ways is to identify biomarkers that correlate with disease susceptibility.

miRNAs are key regulators of a wide variety of biological processes and their presence in sera serve as potential biomarkers of health and disease [13–15]. For example, four serum miRNAs (miR-21-5p, miR-92a-3p, miR-148b-3p, and miR-125a-5p) are novel potential biomarkers for cured pulmonary tuberculosis, with 83.96% accuracy [16]. Another example concerns cervical cancer: MiR-21-5p and miR-34a expression, and human telomerase RNA component (hTERC) amplification, are more specific than human papilloma virus (HPV) positivity in differentiating low-grade from high-grade cervical disorders, which suggests that miR-21-5p, miR-34a and hTERC might serve as surrogate markers for cervical cancer progression [17]. A third example is MiR-182-5p, which may play an important role in the hepatocarcinogenesis of mice exposed to TCE [18]. Additionally, a previous study of TCE-exposed Chinese workers found that seven serum miRNAs showed significant differences between exposed and unexposed workers, with miR-150-5p and let-7b-5p showing significant inverse exposure-response associations with TCE exposure. miR-150-5p is involved in B cell receptor pathways and let-7b-5p plays a role in the innate immune response processes of potential importance in the etiology of non-Hodgkin lymphoma [19]. In this study, we profiled the miRNAs in serum related to THS and evaluated the potential clinical value of differentially expressed miRNAs.

## Methods

### TCE exposure investigation

Occupational TCE exposure factories in Shenzhen were managed on a regional basis, with more than one factory in one area. To understand environmental TCE exposure levels, we selected a Shenzhen city hardware electroplating factory that has no other workplace agents with potential human toxicity. All sample collection was performed according to the national standard “Specifications of air sampling for hazardous substances monitoring in the workplace” (GBZ 159–2004). Briefly, a 2-h air sample was taken at a flow rate of 200 mL/min using an activated carbon tube. Immediately after sampling, the ends of the activated carbon tube were closed and transported in a clean container. The activated carbon in the front and back sections were separately poured into two solvent desorption bottles, and 1.0 mL of carbon disulfide was added to each bottle. After blocking, the mixture was desorbed for 30 min and shaken from time to time. The sample solution was used for measurement. 5 mL water samples were directly collected for detection. For soil samples, a 2 g soil sample was added to 5 mL water for headspace determination. Parallel samples were taken for all samples.

All sample detection was conducted via gas chromatography according to the national standard “Determination of toxic substances in workplace air” (GBZ/T 300.78–2017). Operating conditions of the gas chromatograph (Agilent 6890 N, CA, USA) were as follows: 1.0 μL sample was added to the gasification chamber where the temperature was held at 250 °C. The split ratio was 5:1. The capillary column (Agilent, CA, USA) was 30 m × 0.53 mm × 3.0 μm. Initial temperature of the column was 60 °C, then rose to 80 °C at 10 °C/min, then rose to 140 °C at 20 °C/min. The flow rate of carrier gas (nitrogen) was 4.0 mL/min. The temperature of the detection chamber was 300 °C. For water and soil samples, the temperature was 60 °C and the time was 20 min. The measured peak area values were obtained to calculate the concentration of TCE in the samples.

### Subjects

This study was conducted in accordance with the ethics principles of the Declaration of Helsinki (World Medical Association, 2017) and was approved by the Medical Ethics Committee of Shenzhen Center for Disease Control and Prevention (No. R2018018). All subjects gave their written informed consent. THS patients were diagnosed according to skin rash characteristics, clinical condition and examination indices by the diagnostic criteria of occupational medicamentosa-like dermatitis due to trichloroethylene (GBZ 185–2006) issued by the Ministry of Health of the People’s Republic of China. Between March 2010 and July 2015, 39 patients and 39 age- and sex-matched TCE contacts were enrolled in our study. All subjects were from the same area factories. They all engaged in the degreasing work. According to the basic requirements of the experiments, five patients and five TCE contacts were selected for miRNAs screening, while the other 34 patients and 34 TCE contacts were for miRNAs validation, THS model establishment and clinical correlation analysis. Table S1 shows the detailed characteristics of the two groups.

### Serum collection

All serum samples were collected at the Shenzhen Prevention and Treatment Center for Occupational Diseases. Approximately 4 mL of blood, collected from each subject when admitted to the hospital during the acute, untreated phase of THS, was centrifuged at 4 °C for 10 min to collect serum. Each serum sample was divided into two aliquots: One was used for clinical biochemical detection of liver function; the other was stored at − 80 °C for later analysis.

### miRNAs screening

miRNAs screening was performed with miRCURY LNA™ Universal RT microRNA PCR. Briefly, serum samples were homogenized with 750 μL TRIZOL-LS® Reagent (Invitrogen life technologies, Carlsbad, CA, USA). After homogenization, chloroform was added. The samples were shaken vigorously by hand and then centrifuged at 13000 g 4 °C for 15 min. RNA remained exclusively in the colorless upper aqueous phase, which was then transferred to a fresh tube. Isopropyl alcohol was added and RNA precipitated on the side and bottom of the tube after centrifugation. After washing with 75% ethanol, RNA was dissolved in RNase-free water and incubated for 10 min at 55 °C to 60 °C. The yield and quality of RNA were detected via UV absorbance assay (see Table S2). The template RNA samples were then used to synthesize cDNA followed by Real-time PCR via miRCURY LNA™ Universal RT microRNA PCR chip (Exiqon, Vedbaek, Denmark) in ABI PRISM 7900 Biosystems (Foster, CA, USA). Data were analyzed using GenEx qPCR analysis software (MultiD, Gteborg, Sweden).

### miRNAs validation

Five miRNAs targeting SET (a nuclear proto-oncoprotein negatively regulating protein phosphatase 2A) mRNA differentially expressed in TCE-induced hepatotoxicity (data not shown) were used for the larger sample validation. Experimental procedures were consistent with miRNAs screening. They were both finished by KangChen Bio-tech Co., Ltd. (Shanghai, China). cDNA synthesis was done in 20 μL reaction systems, including 2 μL dNTP, 2 μL 10× RT buffer, 0.3 μL RT primer, 150 ng RNA template, 0.2 μL MMLV reverse transcriptase, 0.3 μL RNase inhibitors and RNase-free water. RT-PCR was preceded at 16 °C for 30 min, 42 °C for 40 min and 85 °C for 5 min in GeneAmp 9700 PCR System (Applied Biosystems, Foster, CA, USA). RT primers and sequences are shown in Tables 1 and 2, respectively. miR-93-5p was used as the internal reference.

**Table 1:** RT primers of 5 significant miRNAs

miRNAs	RT primers 5′-3′
hsa-miR-93-5p ^a^	GTCGTATCCAGTGCGTGTCGTGGAGTCGGCAATTGCACTGGATACGACCTACCTG
hsa-miR-34a-5p	GTCGTATCCAGTGCGTGTCGTGGAGTCGGCAATTGCACTGGATACGACACAACCA
hsa-miR-193b-3p	GTCGTATCCAGTGCGTGTCGTGGAGTCGGCAATTGCACTGGATACGACAGCGGGA
hsa-miR-21-5p	GTCGTATCCAGTGCGTGTCGTGGAGTCGGCAATTGCACTGGATACGACTCAACA
hsa-miR-10a-5p	GTCGTATCCAGTGCGTGTCGTGGAGTCGGCAATTGCACTGGATACGACCACAAA
hsa-miR-339-5p	GTCGTATCCAGTGCGTGTCGTGGAGTCGGCAATTGCACTGGATACGACCGTGAGCTC

^a^Internal reference gene

**Table 2:** Primer sequences of 5 significant miRNAs

miRNAs	Primer sequence 5′-3′
hsa-miR-93-5p ^a^	F: GGCAAAGTGCTGTTCGTG R: CAGTGCGTGTCGTGGAGT
hsa-miR-34a-5p	F:GGGGTGGCAGTGTCTTAGC R:CAGTGCGTGTCGTGGAGT
hsa-miR-193b-3p	F:CGGAACTGGCCCTCAAAG R:CAGTGCGTGTCGTGGAGT
hsa-miR-21-5p	F:GGGGGGTAGCTTATCAGACTG R:CAGTGCGTGTCGTGGAGT
hsa-miR-10a-5p	F:GGGTACCCTGTAGATCCGA R:CAGTGCGTGTCGTGGAGT
hsa-miR-339-5p	F:GGGTCCCTGTCCTCCA R:TGCGTGTCGTGGAGTC

^a^Internal reference gene

### SYBR™ green PCR validation

Serum miRNAs extraction was in accordance with the operating instructions of miRNeasy Serum/Plasma Kit (50) (QIAGEN, Dusseldorf, Germany). Briefly, 200 μL serum was mixed with 700 μL QIAzollysate on ice for 5 min. 150 μL chloroform was added and then centrifuged at 12000×g, 4 °C for 15 min. The supernatant was transferred to a new tube and mixed with 1.5 times volume of anhydrous ethanol. This was transferred to the RNeasy Mini Spin of the Kit to obtain the RNA sample. The RNA samples were detected for purity and concentration with the Nanodrop 2000c (Thermo Fisher, Waltham, MA, USA) and then stored at − 20 °C for further analysis.

According to the instructions of miScript II reverse transcription PCR kit (QIAGEN, Dusseldorf, Germany), 20 μL reverse transcription reaction system was prepared, including 4 μL 5× miScript HiSpecbuffer, 2 μL 10× miScript Nucleics Mix, 2 μL miScript Reverse Transcriptase Mix, 4 μL Template RNA and RNase-free water. This was incubated at 37 °C for 60 min and heated at 95 °C for 5 min. cDNA was obtained and saved at − 20 °C. SYBR™ Green PCR using a GeneAmp 9700 PCR instrument (Applied Biosysterms). 20 μL Real-time PCR reaction system was then prepared, including 4 μL 2× QuantiTect SYBR Green PCR Master Mix, 10 μL 10× miScript Universal Primer, 2 μL 10× miScript Specific Primer, 2 μL Template cDNA and 2 μL RNase-free water. This mixture was centrifuged at 1000 rpm, 4 °C for 1 min. The amplification reaction was then started using the following PCR program: first activated at 95 °C for 15 min, denaturized at 94 °C for 15 s, then annealed at 55 °C for 30 s and exceeded at 70 °C for 34 s. The last 3 steps were for 40 cycles. Resulting data were analyzed using the 2^-ΔΔCt^ method. Three experimental replicates were used for each sample for SYBR™ Green PCR validation.

### Taqman® MGB PCR validation

According to the instructions of TaqMan® microRNA reverse transcription kit (Thermo Fisher), 5 μL reverse transcription reaction system was prepared, including 0.05 μL 100 mM dNTPs, 0.33 μL MultiScribe Reverse Transcriptase, 0.5 μL 10× reverse transcription buffer, 0.063 μL Rnase inhibitor, 1 μL 5× RT primer, 1 μL template RNA and 2.057 μL RNase-free water. This solution was incubated at 16 °C for 30 min, 42 °C for 30 min, and heated at 85 °C for 5 min. cDNA was obtained and stored at − 20 °C. TaqMan® MGB PCR was conducted in 7900HT fluorescence quantitative PCR instrument (Applied Biosysterms).10 μL Real-time PCR reaction system was then prepared, including 5 μL 2× TaqMan Universal PCR Master Mix, 0.5 μL 20× TaqMan miRNA probe, and 4.5 μL template cDNA. This solution was centrifuged at 1000 rpm 4 °C for 1 min. Amplification reaction proceeded as follows: activated at 95 °C for 10 min, then at 95 °C for 15 s, and at 60 °C for 1 min. The 3-step was for 40 cycles. CT value was calculated after the acquisition of fluorescence data at 60 °C. Three experimental replicates was used for each sample in Taqman® MGB PCR validation.

### Model establishment and validation

The diagnostic model of THS was established based on the validated miRNAs via the support vector machine (SVM) algorithm. SVM is based on the principle of structural risk minimization, mainly suitable for small sample problems. Firstly, △CT values of the validated miRNAs in 68 samples (34 THS and 34 controls) was entered into Matlab 2014a software. Secondly, these samples were randomly assigned to a training set and a test set. The training set used to construct the model contained 60% of the samples (22 THS and 18 controls), while the test set containing 40% (12 THS and 16 controls) was used to verify the model. Thirdly, the input vectors of the training set and test set were normalized for easy calculation. Fourthly, the optimal model via SVM algorithm was established by 3 times cross validation. Lastly, the fitting effect of the model was evaluated by the test set.

### Clinical correlation analysis

Correlation analysis was conducted via the Spearman correlation test between validated miRNAs and liver function indices. Liver function was assessed using a DXC800 automatic biochemical analyzer (Beckman, Brea, CA, USA). Indices included total protein (TP), albumin (ALB), globulin (GLB), total bilirubin (TBIL), direct bilirubin (DBIL), indirect bilirubin (IBIL), alanine aminotransferase (ALT), aspartate aminotransferase (AST), glutamyl transpeptidase (GGT) and alkaline phosphatase (ALP).

### Statistical analysis

SPSS 19.0 software was used for statistical analysis. Student’s *t* test was used for analyzing normally distributed continuous variables between two groups, while the Wilcoxon test was used for non-normally distributed continuous variables. Spearman correlation analysis was used to analyze the correlation between miRNAs and liver function indices. *p* < 0.05 was considered statistically significant.

## Results

### TCE exposure and clinical characteristics of THS patients

As shown in Table 3, we found that there were TCE in the samples of air, water and soil. TCE air concentration near the workshop door, the treasury, and the sewage pool exceeded those in other places in the factory. THS patients became ill about 1 month after commencement of exposure while TCE contacts without onset even though exposed for a longer time (Table S1). The clinical characteristics included fever, rash, superficial lymph node enlargement, and liver damage and so on (Table S3).

**Table 3:** TCE concentrations near a hardware electroplating factory in Shenzhen

Sample type	Sources	Concentrations
Air (mg/m^3^)		
	Rooftop exhaust one	0.059
	Rooftop exhaust two	0.057
	In the workshop	2.710
	Workshop door	0.354
	Warehouse door	0.393
	Sewage pool	0.713
	Road one outside of factory	0.075
	Road two outside of factory	0.083
Water (μg/L)		
	Sewage outlet	8.409
	River near the sewage outlet	3.909
Soil (μg/kg)		
	Flower bed on the window of workshop	0.244
	Flower bed three meters outside the workshop door	0.325

“workshop” refer to the workshop where TCE was used

### Expression profile of miRNAs

A total of 69 miRNAs was significantly expressed between THS patients and TCE contacts via miRCURY LNA™ miRNA PCR chip (Showed in Table S4). Compared with the TCE contacts, 30 miRNAs were down-regulated and 39 up-regulated, as shown in supplementary Fig. S1.

### Validation of miRNAs

Among the 69 miRNAs, we selected five miRNAs targeting SET mRNA differentially expressed in TCE-induced hepatotoxicity (data not shown) for further validation. These included: hsa-miR-34a-5p (miR-34a), hsa-miR-193b-3p (miR-193b), hsa-miR-21-5p (miR-21), hsa-miR-10a-5p (miR-10a) and hsa-miR-339-5p (miR-339). Large-sample validation showed these five miRNAs were highly expressed in THS patients and significant different from TCE contacts (controls). The fold changes in THS patients were 4.95, 10.56, 3.87, 1.55 and 1.90, respectively.

**Fig. 1:**
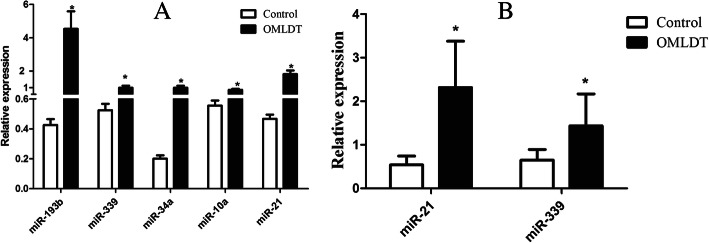
Differential expression of miRNAs in 34 THS patients and 34 TCE contacts

We further verified the expressions of these five miRNAs by SYBR™ Green PCR and Taqman® MGB PCR in 34 THS patients (THS) and 34 TCE contacts (controls). SYBR™ Green PCR showed that these five miRNAs were also statistically significant and highly-expressed in THS. However, after Taqman® MGB PCR validation, only miR-339 and miR-21 were statistically significant (Fig. 1).

### THS diagnostic model

**Fig. 2:**
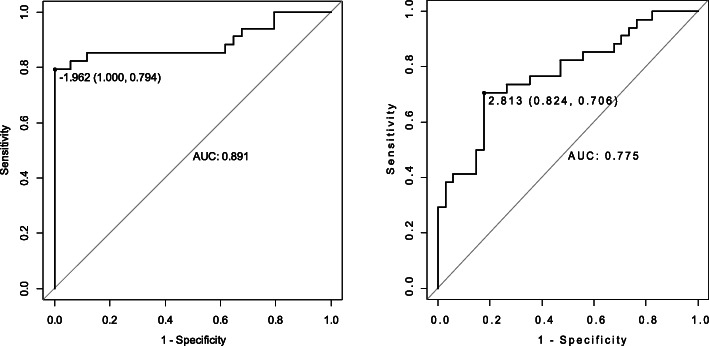
ROC curves of miR-21 and miR-339

Receiver-operating characteristic (ROC) curve measures discrimination was used to classify individuals with or without THS. The closer the area under the curve (AUC) value is to 1.0, the stronger the classification. The closer the AUC value is to 0.5, the weaker the classification. The AUC values of miR-21 and miR-339 were 0.891 and 0.775, respectively (Fig. 2). MiR-21 and miR-339 had good discrimination power between THS patients and TCE contacts.

We then established the diagnostic model via Matlab 2014a software to discriminate THS patients from TCE contacts by SVM algorithm. To verify the accuracy of the THS model established with miR-21 and miR-339, we tested 28 samples including 12 THS and 16 controls. The model correctly classified 12/12 THS patients and 12/16 TCE contacts. The sensitivity and specificity were 100 and 75%, respectively. The overall accuracy was 86%, indicating good separating capacity.

### Clinical correlation analysis

We performed a correlation analysis of liver function indicators to relate the identified miRNAs to clinical parameters. We first detected liver function-related indicators in 68 validated samples (Table S5). TP, ALB and GLB values were significantly decreased in THS patients while TBIL, DBIL, IBIL, ALT, AST, GGT and ALP values were significantly increased. Using the indicators and △CT values of miR-21 and miR-339, we then conducted a correlation analysis between miRNAs and liver function indicators (Table 4). MiR-21 and miR-339 were both negatively correlated with TP, ALB and GLB values. Moreover, miR-21 was positively associated with values for TBIL, DBIL, ALT, AST and GGT.

**Table 4:** Correlation analysis of liver function indices with miR-21 and miR-339

miRNAs	Index	TP	ALB	GLB	TBIL	DBIL	IBILI	ALT	AST	GGT	ALP
miR-21	r^a^	−0.524	−0.490	− 0.398	0.302	0.295	0.224	0.307	0.240	0.366	0.117
	*p*	0.000	0.000	0.001	0.012	0.015	0.066	0.011	0.049	0.002	0.340
miR-339	r^a^	−0.428	−0.316	−0.360	0.009	0.018	0.079	0.156	0.027	0.218	0.040
	*p*	0.000	0.009	0.009	0.943	0.883	0.520	0.203	0.824	0.075	0.747

^a^Correlation analysis was conducted via the Spearman correlation test between validated miRNAs (miR-21 and miR-339) and liver function indices of 34 patients and 34 TCE contacts

## Discussion

TCE has become a key occupational health threat in Guangdong Province that has brought major economic losses. At the same time, TCE is discharged into the environment through volatilization, container leakage, waste liquid and burning of chlorinated organic products, resulting in increasingly a serious environmental pollution problem [20]. The concentration of TCE in the factory of Shenzhen city was obviously higher (0.6 mg/m^3^ on average) than in Guangzhou city (2.1 μg/m^3^) [21]. In the Shenzhen factory studied here, we found that airborne TCE near a metal electroplating workshop and sewage pool were higher than the rest of the factory. The content of TCE in water (6.1 μg/L on average) was lower than that of Puerto Rico (43.6 μg/L) [22] while closer to that of the USA (3.8 μg/L) [23]. Taken in concert, the impact of occupational and environmental exposure to TCE in air has become a public health issue of general concern.

THS is a dose-independent and potentially life-threatening disease [24]. In our study, the airborne TCE concentration of cases was slightly higher than that in the environment, but was lower than in other places. It should be noted, however, the occupational exposure level of the cases judging from the biological monitoring results was much higher than expected from our environmental monitoring data [25, 26]. By the government involved in the area, the usage of TCE was strictly controlled when we detected its use. Nonetheless, in the occupational settings that THS patients and TCE contacts did the degreasing work in the factories, the patients became ill within an average of 1 month of exposure. But the TCE contacts didn’t get sick even though exposed for a longer time. The occupational exposure duration and amounts between them were different. This suggested that there were individual differences in THS disease.

Occupational exposure to TCE is known to be associated with differential expression of certain serum microRNAs [19, 27]. In this study, we used microRNA microarray technology to screen and identify 69 significantly expressed serum microRNAs. Five microRNAs (hsa-miR-193b-3p, hsa-miR-10a-3p, hsa-miR-339-5p, hsa-miR-34a-5p and hsa-miR-21-5p) were verified by the SYBR™ Green PCR method. Further, hsa-miR-339-5p and hsa-miR-21-5p were verified by Taqman® MGB fluorescence quantitative PCR. They were both up-regulated in the TCE-exposed group. These results suggested that miR-339-5p and miR-21-5p are associated with THS. However, because of the limited number of screened miRNAs and small sample size, we need to study miRNA expression profiles with independent validation in a larger cohort of participants.

Support Vector Machine (SVM) algorithms are commonly used in the classification of small samples. In blood samples taken from 20 patients with multiple sclerosis, a total of 48 miRNAs assessed by radial basis function kernel SVM and 10-fold cross validation yielded a specificity of 95%, a sensitivity of 97.6%, and an accuracy of 96.3% [28]. The diagnostic model of multiple sclerosis established by the expression of miR-145 can better distinguish multiple sclerosis patients and healthy people with a specificity of 89.5%, a sensitivity of 90.0%, and an accuracy of 89.7%. In our study, the optimal parameters of the THS model via SVM algorithm were obtained by 3-fold cross validation. All THS patients were correctly predicted. Twelve out of 16 TCE controls were also correctly validated. The overall accuracy rate was 86%. The model established on the basis of miRNA-21 and miRNA-339 by these parameters had a good fitting effect.

THS is often associated with liver damage. The concentrations of TP, ALB and GLB in the blood of patients were lower than that of the control, while the concentrations of TBIL, DBIL, IBILI, ALT, AST, GGT and ALP were higher than those of the control. Correlation analysis between liver function indices and the two miRNAs found that both miR-21 and miR-339 were both negatively correlated with TP, ALB and GLB. The relative expression of miR-21-5p was positively correlated with TBIL (*r* = 0.302), DBIL (*r* = 0.295), ALT (*r* = 0.307), AST (*r* = 0.240) and GGT (*r* = 0.366), but miR-339-5p showed no correlation. For miR-21-5p, target genes are involved in important life processes such as cytoskeleton formation, growth inhibition, apoptosis, cell differentiation and cell metabolism [29, 30]. MiR-21-5p inhibited mitogen-activated protein kinase-kinase 3 (MAP 2 K3) to promote malignant proliferation of hepatocytes, and it may play an important role in tumor migration and infiltration regulation [31]. MiR-339-5p is closely related to cell apoptosis and growth inhibition processes, which are involved in tumor growth inhibition [32, 33]. miR-339-5p expression in hepatocellular carcinoma (HCC) was significantly lower than that in normal liver tissue [34]. The expression level of miR-339-5p was related to the prognosis of HCC patients, and the high expression of miR-339-5p could inhibit the invasion of hepatocarcinoma cells.

THS is an immune-related disease. We found 18 biological processes related to the immune response via target gene prediction of the two miRNAs (Fig. S2). The target genes of miR-21 and miR-339 were predicted by miRDB database (http://mirdb.org/). These genes were then analyzed by an R package (4.0.3 version) named “clusterProfiler” [35] for functional enrichment. While our results may provide clues to the pathogenesis of THS, our study has limitations since we don’t known whether they are specific for THS or also found in other liver disorders.

## Conclusion

In conclusion, this limited study of miRNAs profiles in the sera of THS patients differs from that of controls: expression profiles for miR-21-5p and miR-339-5p were associated with THS. Additional studies that include a larger sample size and subjects with a range of liver disorders including THS are needed to place the present findings in perspective. However, our findings may provide potential indicators of THS pathogenesis and support new approaches for human health risk assessment.

## Supplementary Information

**Additional file 1: Table S1.** Demographic characteristic of the THS patients and TCE contacts. **Table S2.** RNA yield and quality assessment in 10 serum samples. **Table S3.** The clinical characteristics of 39 THS patients. **Table S4.** 69 differentially expressed miRNAs between THS patients and TCE contacts. **Table S5** Liver functions of THS patients and TCE contacts. **Fig. S1.** Heat map and hierarchical clustering of differential miRNAs. **Fig. S2.** The target genes prediction of miR-21 and miR-339

## Data Availability

Please contact author for data requests.
