# Correction to: Serum levels of miR-21-5p and miR-339-5p associate with occupational trichloroethylene hypersensitivity syndrome

**DOI:** 10.1186/s12995-021-00315-1

**Published:** 2021-08-11

**Authors:** Wei Liu, Jian Zheng, Xiaohu Ren, Yuxuan Xie, Dafeng Lin, Peimao Li, Yuan Lv, Maggie Pui Man Hoi, Yanfang Zhang, Jianjun Liu

**Affiliations:** 1 Shenzhen Key Laboratory of Modern Toxicology, Shenzhen Medical Key Discipline of Health Toxicology (2020-2024), Shenzhen Center for Disease Control and Prevention, Shenzhen, 518055 China; 2 State Key Laboratory of Quality Research in Chinese Medicine, Institute of Chinese Medical Sciences, University of Macau, Macau, China; 3 Key Laboratory of Molecular Epidemiology of Hunan Province, School of Medicine, Hunan Normal University, Changsha, 410081 China; 4 Shenzhen Prevention and Treatment Center for Occupational Diseases, Shenzhen, 518020 China

**Correction to: J Occup Med Toxicol 16, 19 (2021)**

**https://doi.org/10.1186/s12995-021-00308-0**

Following the publication of the original article [1], we were notified that the second and the fourth affiliation should be swapped. Descriptions for Figs. 1 and 2 have also been added.

The original article has been corrected.
