# Supplementary material for: Serum levels of miR-21-5p and miR-339-5p associate with occupational trichloroethylene hypersensitivity syndrome
Source: J Occup Med Toxicol. 2021 May 17;16:19. doi: 10.1186/s12995-021-00308-0 (PMC8127200; doi:10.1186/s12995-021-00308-0)
Supplement: Supplementary file 1 — Additional file 1: Table S1. Demographic characteristic of the THS patients and TCE contacts. Table S2. RNA yield and quality assessment in 10 serum samples. Table S3. The clinical characteristics of 39 THS patients. Table S4. 69 differentially expressed miRNAs between THS patients and TCE contacts. Table S5 Liver functions of THS patients and TCE contacts. Fig. S1. Heat map and hierarchical clustering of differential miRNAs. Fig. S2. The target genes prediction of miR-21 and miR-339 [file 12995_2021_308_MOESM1_ESM.docx]

**Supporting information Legend**

**Table S1** Demographic characteristic of the THS patients and TCE contacts

**Table S2** RNA yield and quality assessment in 10 serum samples

**Table S3** The clinical characteristics of 39 THS patients

**Table S4** 69 differentially expressed miRNAs between THS patients and TCE contacts

**Table S5** Liver functions of THS patients and TCE contacts

**Figure S1** Heat map and hierarchical clustering of differential miRNAs.

**Figure S2** The target genes prediction of miR-21 and miR-339


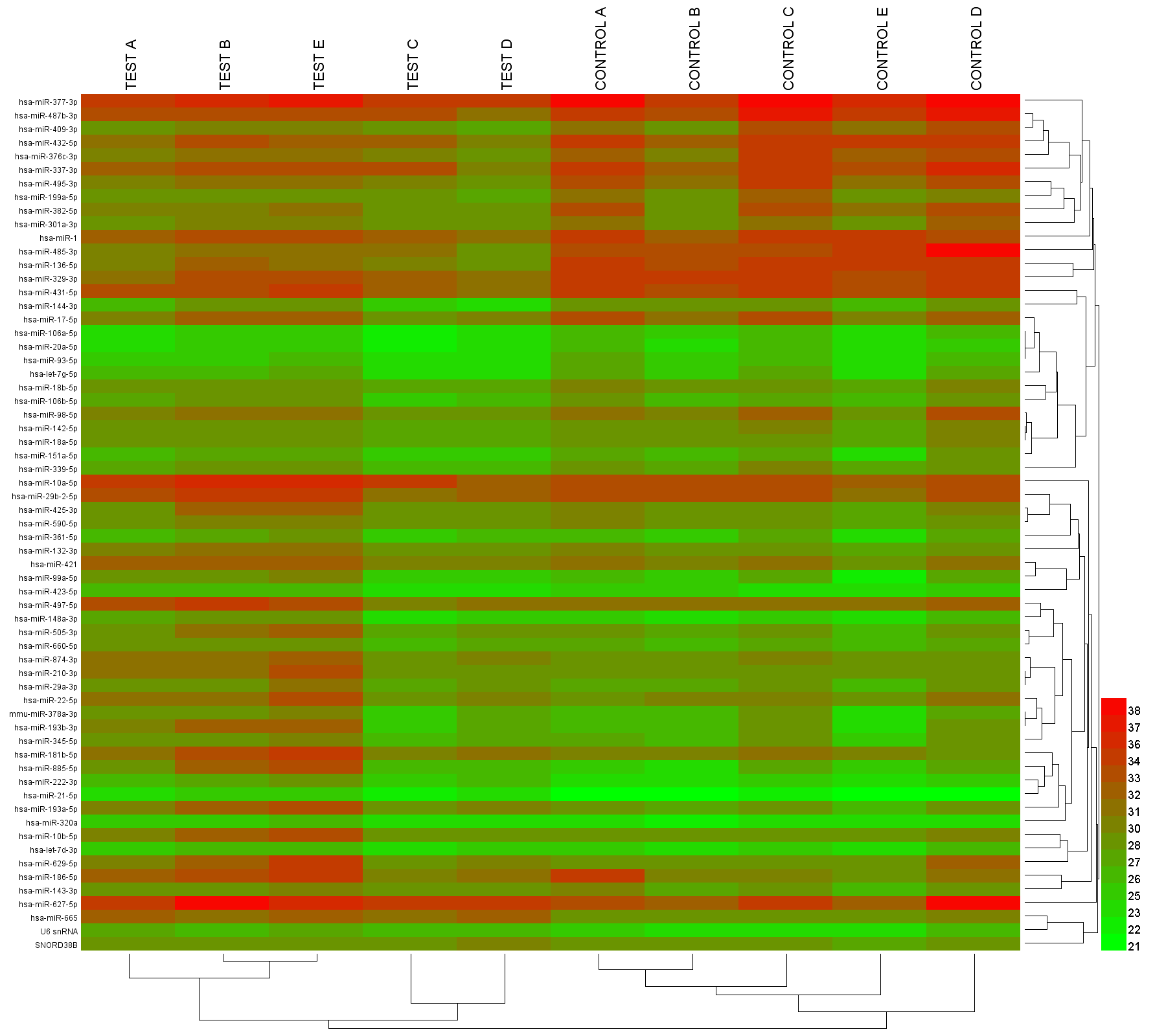


**Figure S1** Heat map and hierarchical clustering of differential miRNAs. The heat map indicates the results of a two-way hierarchical clustering of miRNAs and samples. The upper test A, B, C, D, E represent the THS patient samples, while the control A, B, C, D, E represent the TCE contact samples. Each row represents a miRNA and each column represents a sample. The miRNA clustering tree is shown on the right. The color scale displays the relative expression level of a miRNA in the certain slide: red, high relative expression level; green, low relative expression level.


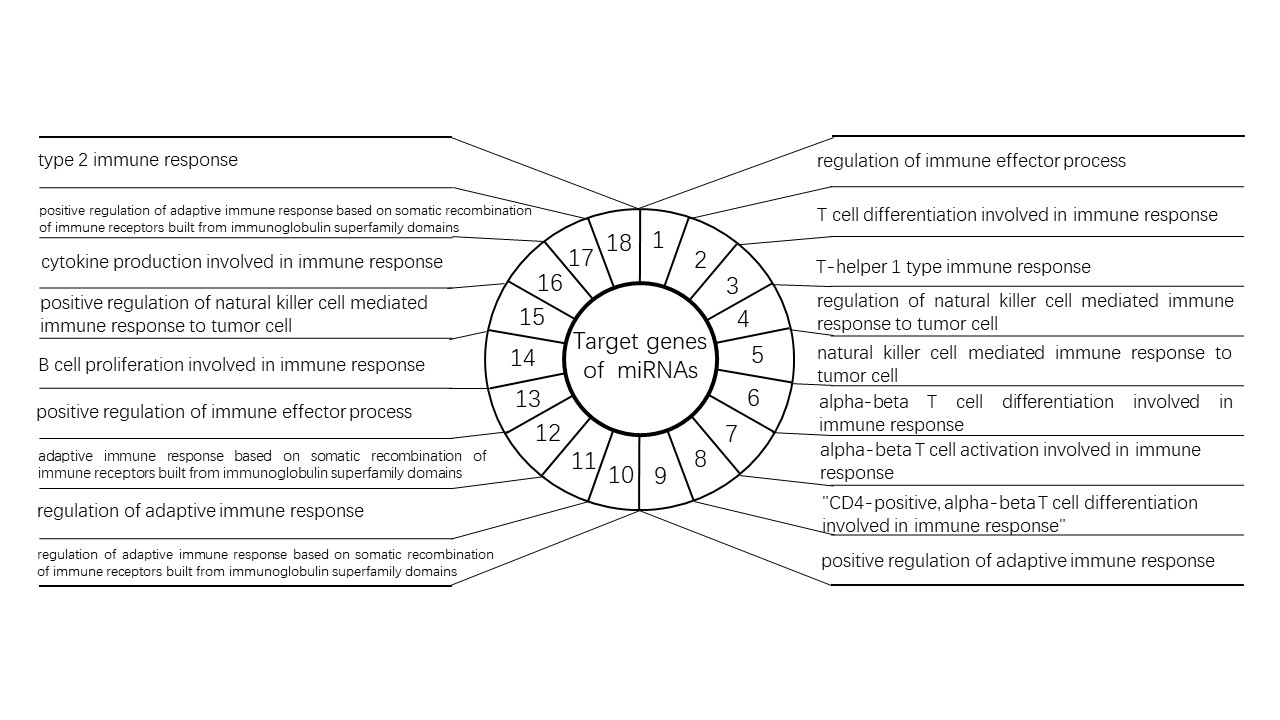


**Figure S2** The target genes prediction of miR-21 and miR-339

**Table S1** Demographic characteristic of the THS patients and TCE contacts ($\overline{X}$±SEM)

| Groups | Items | Total |
| --- | --- | --- |
| THS patients | Male / Female | 26 / 13 |
|  | Average age (Years) | 25.5±6.2 |
|  | Exposure period (Working days) | 31.1±2.2*** |
| TCE contacts | Male / Female | 25 / 14 |
|  | Average age (Years) | 25.9±9.3 |
|  | Exposure period (Working days) | 562.1±101.2 |

*** *p*< 0.001 vs TCE contacts.

**Table S2** RNA yield and quality assessment in 10 serum samples

| Sample | OD_260_ | OD_280_ | OD_260_/OD_280_ | RNA concentration (μg/μL) |
| --- | --- | --- | --- | --- |
| Patient 1 | 1.2 | 0.7 | 1.7 | 48.8 |
| Patient 2 | 1.0 | 0.6 | 1.6 | 41.6 |
| Patient 3 | 1.1 | 0.8 | 1.4 | 43.1 |
| Patient 4 | 0.8 | 0.5 | 1.6 | 32.2 |
| Patient 5 | 1.0 | 0.6 | 1.7 | 40.8 |
| TCE contact 1 | 1.6 | 0.9 | 1.7 | 62.1 |
| TCE contact 2 | 1.6 | 1.0 | 1.6 | 62.8 |
| TCE contact 3 | 1.5 | 1.0 | 1.5 | 60.3 |
| TCE contact 4 | 1.6 | 0.9 | 1.7 | 61.8 |
| TCE contact 5 | 1.4 | 0.8 | 1.7 | 57.4 |

**Table S3** The clinical characteristics of 39 THS patients

| Symptoms | Headache | Appetite decrease | Fatigue | Vomiting | Fever |
| --- | --- | --- | --- | --- | --- |
| Positive patients | 12 | 31 | 15 | 10 | 33 |
| Positive rate (%) | 30.8 | 79.5 | 38.5 | 25.6 | 84.6 |
| signs | Rash | Lymph node enlargement | Eyes lesion | Liver damage |  |
| Positive patients | 39 | 24 | 22 | 32 |  |
| Positive rate (%) | 100 | 61.5 | 56.4 | 82.1 |  |

**Table S4** 69 differentially expressed miRNAs between THS patients and TCE contacts

| miRNAs | *p* | Change Folds |
| --- | --- | --- |
| Up-regulated miRNAs | | |
| has-miR-885-5p | 0.04 | 12.86 |
| has-miR-193a-5p | 0.00 | 8.72 |
| has-miR-203a | 0.02 | 7.71 |
| has-miR-34a-5p § | 0.03 | 7.46 |
| has-miR-193b-3p § | 0.02 | 6.90 |
| has-miR-665 | 0.01 | 5.77 |
| has-miR-21-5p § | 0.02 | 5.47 |
| has-miR-181b-5p | 0.02 | 5.33 |
| has-miR-222-3p | 0.01 | 4.67 |
| U6 snRNA | 0.00 | 4.61 |
| has-miR-99a-5p | 0.04 | 4.41 |
| has-miR-505-3p | 0.00 | 4.26 |
| has-miR-452-5p | 0.04 | 4.24 |
| mmu-miR-378a-3p | 0.03 | 4.13 |
| has-miR-210-3p | 0.02 | 3.75 |
| has-miR-627-5p | 0.03 | 3.57 |
| has-miR-629-5p | 0.04 | 3.54 |
| has-miR-148a-3p | 0.04 | 3.31 |
| has-miR-10b-5p | 0.04 | 3.18 |
| has-miR-29a-3p | 0.02 | 3.12 |
| has-miR-149-5p | 0.04 | 2.97 |
| has-miR-320a | 0.02 | 2.87 |
| has-miR-10a-5p § | 0.02 | 2.81 |
| SNORD38B | 0.03 | 2.76 |
| has-miR-660-5p | 0.00 | 2.71 |
| has-miR-874-3p | 0.02 | 2.71 |
| has-miR-22-5p | 0.04 | 2.63 |
| has-miR-497-5p | 0.02 | 2.58 |
| has-miR-345-5p | 0.03 | 2.54 |
| has-miR-132-3p | 0.03 | 2.46 |
| has-miR-421 | 0.00 | 2.45 |
| has-miR-186-5p | 0.03 | 2.30 |
| has-miR-425-3p | 0.01 | 2.25 |
| has-miR-423-5p | 0.03 | 2.02 |
| has-let-7d-3p | 0.03 | 1.89 |
| has-miR-143-3p | 0.04 | 1.72 |
| has-miR-29b-2-5p | 0.01 | 1.69 |
| has-miR-590-5p | 0.01 | 1.64 |
| has-miR-361-5p | 0.03 | 1.61 |
| Down-regulated miRNAs | | |
| hsa-miR-106b-5p | 0.04 | -1.28 |
| hsa-miR-151a-5p | 0.03 | -1.47 |
| hsa-let-7g-5p | 0.01 | -1.53 |
| hsa-miR-142-5p | 0.00 | -1.60 |
| hsa-miR-93-5p | 0.01 | -1.72 |
| hsa-miR-17-5p | 0.01 | -1.73 |
| hsa-miR-18a-5p | 0.00 | -1.81 |
| hsa-miR-18b-5p | 0.00 | -1.86 |
| hsa-miR-20a-5p | 0.00 | -1.89 |
| hsa-miR-106a-5p | 0.00 | -1.97 |
| hsa-miR-339-5p § | 0.01 | -2.04 |
| hsa-miR-431-5p | 0.04 | -2.08 |
| hsa-miR-98-5p | 0.00 | -2.12 |
| hsa-miR-144-3p | 0.02 | -2.52 |
| hsa-miR-377-3p | 0.01 | -2.71 |
| hsa-miR-301a-3p | 0.01 | -2.77 |
| hsa-miR-1 | 0.04 | -2.86 |
| hsa-miR-337-3p | 0.04 | -2.88 |
| hsa-miR-199a-5p | 0.01 | -3.14 |
| hsa-miR-301b | 0.01 | -3.61 |
| hsa-miR-382-5p | 0.01 | -3.71 |
| hsa-miR-432-5p | 0.03 | -3.90 |
| hsa-miR-495-3p | 0.00 | -4.05 |
| hsa-miR-376b-3p | 0.01 | -4.08 |
| hsa-miR-376c-3p | 0.00 | -4.15 |
| hsa-miR-329-3p | 0.01 | -4.65 |
| hsa-miR-409-3p | 0.01 | -5.09 |
| hsa-miR-487b-3p | 0.01 | -6.25 |
| hsa-miR-136-5p | 0.00 | -9.09 |
| hsa-miR-485-3p | 0.01 | -12.22 |

§ These miRNAs were selected for larger sample validation.

**Table S5** Liver functions of THS patients and TCE contacts ($\overline{X}$±SEM)

| Groups | TP | ALB | GLB | TBIL | DBIL | IBILI | ALT | AST | GGT | ALP |
| --- | --- | --- | --- | --- | --- | --- | --- | --- | --- | --- |
| THS | 59.3±8.1 * | 34.0±4.8 * | 29.1±21.4 * | 45.1±59.7 * | 25.0±31.9 * | 20.1±28.6 * | 385.4±401.9 * | 183.8±349.2 * | 186.3±152.5 * | 157.3±93.3 * |
| Control | 74.0±4.2 | 44.1±3.9 | 29.2±6.1 | 9.5±2.3 | 4.3±1.9 | 5.9±1.9 | 18.7±10.7 | 19.2±4.7 | 21.1±10.2 | 93.4±53.8 |

* *p*< 0.05 vs TCE contacts. TP, total protein; ALB, albumin; GLB, globulin; TBIL, total bilirubin; DBIL, direct bilirubin; IBIL, indirect bilirubin; ALT, alanine aminotransferase; AST, aspartate aminotransferase; GGT, glutamyl transpeptidase; ALP, alkaline phosphatase.
